# Asymmetric impact of the 2012 Occupational Health and Safety Law on occupational injury and disease reporting in Türkiye: An interrupted time series analysis with emphasis on emerging female occupational disease patterns

**DOI:** 10.5271/sjweh.4331

**Published:** 2026-11-01

**Authors:** Funda Ekimci Deniz, Zehra Nur Töreyin, Metin Akgün

**Affiliations:** 1 Department of Family Medicine, Faculty of Medicine, Aydın Adnan Menderes University, Aydın, Türkiye.; 2 Department of Pulmonary Diseases, Faculty of Medicine, Ege University, İzmir, Türkiye.; 3 Department of Pulmonary Diseases, Faculty of Medicine, Ağrı İbrahim Çeçen University, Ağrı, Türkiye.

**Keywords:** engineered stone silicosis, female worker, gender bias, musculoskeletal disorder, occupational disease surveillance, pneumoconiosis, toxic polyneuropathy, worker compensation

## Abstract

**Objectives:**

This study aimed to characterize 18-year trends in occupational injury (OI) and recognized occupational disease (OD) in Türkiye’s compensation-based surveillance, with emphasis on emerging female disease patterns and on the impact of the 2012 Occupational Health and Safety (OHS) Law.

**Methods:**

We extracted nationwide annual data (2007–2024) from Social Security Institution Statistical Yearbooks. We computed rates per 100 000 insured, the OI/OD ratio, and sex-stratified International Classification of Diseases, 10^th^ Revision (ICD-10) sub-code distributions. Segmented regression with two intervention points used Newey–West heteroscedasticity- and autocorrelation-consistent (HAC) standard errors.

**Results:**

The proportion of recognized OD among women rose from 0.9% (2007) to 25.5% (2024) but with marked asymmetry: respiratory OD remained 96% male while nervous-system OD shifted from 24% female (2018) to 75% female (2024), driven by ICD-10 G62.2 toxic polyneuropathy (26 of 40 female cases in 2024). Female musculoskeletal OD rose from 20% to 34% with dominant upper-extremity repetitive-motion sub-codes; no female silicosis was recorded 2018–2024. Annual OI rose 9.1-fold (80 602 to 733 646); recognized OD declined (1208 to 888). The 2012 OHS Law produced an OI level change of +584 per 100 000 (P<0.001) but no statistically significant OD level change (P=0.32); the OI/OD ratio rose from 67 to 826.

**Conclusions:**

The convergence of women’s share of OD toward 25% masks marked diagnostic asymmetries: women are visible primarily through feminized service-sector and chemical-exposure categories and scarcely in pneumoconioses. Reform priorities include diagnosis-stage notification, active screening of female workers in mixed-exposure environments, and gender-sensitive occupational history-taking.

Occupational injury and disease surveillance is a foundational instrument of public health policy. Reliable longitudinal data enable identification of high-risk industries, evaluation of regulatory interventions, and targeting of preventive resources (1, 2). The validity of surveillance systems is constrained by what they measure: occupational injuries are acute, observable, and tied to legally mandated incident reporting, while occupational diseases are often chronic, latent, non-pathognomonic, and dependent on physician-initiated diagnosis (3, 4).

Among the most consequential blind spots of conventional surveillance is gender. A substantial literature documents that workers’ compensation systems systematically reject a higher proportion of female applications, that women face longer diagnostic delays than men with comparable exposures, and that exposure assessments remain calibrated to male-dominated occupations (5–11). Criado Perez (12) has summarized the structural problem: most occupational exposure thresholds and personal protective equipment standards have been calibrated to male reference bodies, rendering female workers invisible to surveillance systems. As female labor force participation expands in countries undergoing sectoral transformation, the question of whether female occupational morbidity becomes visible in compensation-based surveillance – and which diagnostic categories admit them – becomes a primary indicator of system performance.

Türkiye applies two related but distinct statutory definitions. The Occupational Health and Safety Law (Law 6331) uses a broad, prevention-oriented definition – “a disease arising from exposure to occupational risks” – whereas Social Insurance Law 5510 defines occupational disease for insurance purposes as a temporary or permanent disease or bodily or mental impairment caused by repeated factors related to the nature of the insured person’s work or by the conditions under which the work is performed (13, 14). For the national Social Security Institution (Sosyal Güvenlik Kurumu, SGK) recognition, an authorized health service provider prepares a health-board report and supporting medical documents; the final work-relatedness determination is made by the SGK Health Board, with workplace investigation where required (15). SGK yearbooks capture cases recorded after this administrative determination, not all suspected or clinically diagnosed disease. They do not publish the number of applications, rejected claims, reasons for rejection, or awards of specific benefits. The data therefore describe administrative recognition within the social-insurance system rather than clinical incidence.

Against this institutional background, the 2012 OHS Law provides a clear policy breakpoint for examining whether employer-driven injury reporting and the more complex occupational-disease recognition pathway responded differently to regulatory reform. Enacted in December 2012, Law 6331 expanded employers’ responsibilities for recording and reporting occupational injuries and diseases (13). Employers must notify SGK within three working days of learning of an occupational disease, while authorized healthcare providers must notify diagnosed cases within ten days. Suspected cases identified by workplace physicians or other healthcare providers are referred to authorized providers for further assessment. These notification duties coexist with, but do not replace, the separate SGK administrative recognition pathway described above.

The distinction between clinical suspicion, notification, and administrative recognition is particularly relevant to occupational respiratory disease in Türkiye. The country experienced a well-documented silicosis epidemic among denim-sandblasting workers during 2005–2008, followed by the prohibition of denim sandblasting in 2009 (16, 17). More recently, the international emergence of engineered-stone-associated silicosis, with men comprising more than 95% of reported cases in several identified clusters, has highlighted the continuing limitations of occupational-disease surveillance in mixed-exposure work environments (18, 19). A documented Turkish case involving a female worker whose silicosis diagnosis was delayed for ten years because her occupational exposure was not initially elicited illustrates how gendered assumptions in occupational history-taking may impede recognition (6). It also raises the question of how many women working in mixed-exposure roles remain unrecognized in compensation-based surveillance.

In this study, we conducted an 18-year analysis (2007–2024) of national SGK surveillance data with particular attention to female occupational disease patterns. We aim to: (i) describe long-term trends in OI and recognized OD with sex disaggregation; (ii) quantify the impact of the 2012 OHS Law on these surveillance streams using interrupted time series analysis with a secondary intervention point at the COVID-19 pandemic onset; (iii) characterize sex-disaggregated trajectories of major OD diagnostic categories at International Classification of Diseases, 10^th^ Revision (ICD-10) sub-code resolution; and (iv) discuss implications for the recognition of female occupational disease and corresponding policy reform priorities.

## Methods

### Study design and data sources

This is a descriptive and quasi-experimental study of nationwide administrative surveillance data. We extracted annual aggregated counts from Bölüm 3 (Section 3: Occupational Injuries and Occupational Diseases) of the SGK Statistical Yearbooks for 2007 through 2024 (20). The denominator population was the average annual number of active insured persons under Social Insurance Law 5510, Article 4-1/a (employees working under a service contract). Coverage under 4-1/a is not restricted to permanent employment contracts. Self-employed persons (Article 4-1/b) and civil servants (Article 4-1/c) were outside this dataset; 4-1/b workers were additionally excluded because of limited historical comparability.

### Turkish occupational-disease recognition and insurance

The implementing regulation contains a statutory occupational-disease list in five groups: chemical agents, occupational skin diseases, pneumoconioses and other occupational respiratory diseases, occupational infectious diseases, and diseases caused by physical agents. The system is not strictly closed. The list specifies disease-specific minimum exposure and post-exposure liability periods where applicable; there is no universal minimum exposure or single liability period. A disease outside the list, or a listed disease presenting beyond its liability period, may still be recognized when the clinical and laboratory diagnosis and workplace exposure are documented, subject to approval by the Social Insurance High Health Board (15).

Workplace physicians and other healthcare providers who make a preliminary occupational-disease diagnosis refer the worker to an authorized provider. Ministry of Health occupational-disease hospitals and training and research hospitals, and state university hospitals, may issue the health-board report; the final causal determination is made by the SGK Health Board after review of medical evidence and, when considered necessary, a workplace investigation. Official SGK guidance identifies employment and shift records, entry and periodic medical examinations, the health-board report, and supporting clinical tests among the required documents (15). The published yearbooks do not show whether exposure documentation was incomplete or difficult to obtain by disease, occupation, or sex.

Occupational-disease insurance is not limited to permanent employees and does not require a minimum contribution period. Formally employed foreign nationals with a valid work permit are generally subject to Law 5510, subject to bilateral social-security agreements; workers who are unregistered or working without authorization are not represented in SGK records (14). The yearbooks do not cross-classify nationality or migrant status by sex.

Statutory benefits are paid by SGK rather than directly by the employer, although employer recourse or civil liability may arise separately. Temporary incapacity benefit is paid for each medically certified day at one-half of daily earnings during inpatient treatment and two-thirds during outpatient treatment. Permanent incapacity income requires an SGK-assessed loss of occupational earning capacity of ≥10%; for total incapacity it is calculated as 70% of monthly earnings and for partial incapacity it is prorated by the assessed degree (15). The 10% threshold concerns eligibility for permanent income, not the determination of occupational disease itself. Permanent income is not fixed to a predetermined duration; it may be increased, reduced, or discontinued following SGK control examination (20). There is no single universal disease-claim window: notification is due within three working days after the employer or covered self-employed worker learns of the disease; after employment ends, disease-specific liability periods apply, with exceptional review by the Social Insurance High Health Board.

### Operational definition of recognized occupational disease

The SGK Statistical Yearbooks enumerate insured workers recorded by SGK as having an occupational disease following the administrative determination process; earlier yearbooks describe these as *işlemi tamamlanan vakalar* (cases with completed administrative procedures). We therefore operationalized recognized OD as an SGK-determined occupational-disease case recorded in the relevant yearbook. Suspected or clinically diagnosed cases that did not complete the SGK determination pathway are not represented in the dataset. Throughout this article, OD or recognized OD refers to these SGK-recorded cases.

### Variables and data extraction

Primary outcomes were annual counts of reported occupational injuries, recognized occupational diseases, and deaths in each category. Secondary variables included sex disaggregation, distribution by economic activity classified using the Statistical Classification of Economic Activities in the European Community (NACE Rev. 2.1), and detailed sex-disaggregated diagnostic categories at ICD-10 chapter and sub-code level. The diagnostic classification changed in 2014 from the SGK five-group system to ICD-10. Diagnostic-detail tables count distinct codes per case and may exceed summary-table totals; we therefore treat category proportions and within-category sex shares as the most reliable signals. Data extraction used a custom Python parser that auto-detected variable column ordering across years; all values were cross-validated against published totals.

### Statistical analysis

We computed crude rates per 100 000 insured persons. Annual percent change (APC) was computed as APC=(ending/starting)^(1/n) − 1 (21). For interrupted time series analysis, we used segmented linear regression with two intervention points: January 2013 (first full year following the December 2012 OHS Law) and March 2020 [World Health Organization (WHO) declaration of COVID-19]. We selected the two intervention points a priori and excluded other candidates before inspecting the data, in accordance with the standard that ITS impact models should be articulated before fitting (22). March 2020 coincides with the WHO COVID-19 declaration, after which labor markets, employer reporting and SGK Health Committee operations underwent documented disruption. Four further dates were excluded: the 2009 denim-sandblasting ban, whose male-dominated clusters lacked aggregate impact; the 2014 ICD-10 implementation, with sub-code reporting already operative throughout the window; the 2018 engineered-stone silicosis emergence, too small to alter annual OD counts; and the 2020–2021 SGK registration expansions, being incremental operational adjustments. One breakpoint was rejected because a single intervention cannot capture the post-2020 OI/OD divergence; three or more were rejected on degrees-of-freedom grounds, as each additional breakpoint consumes two degrees of freedom and, with T=18, would force at least one segment below the floor for reliable ITS estimation (23). The Newey–West heteroscedasticity- and autocorrelation-consistent (HAC) estimator (24) used a lag truncation of h=1 because at T=18 the lag-1 autocovariance dominates and higher-order terms carry high variance, consistent with h=⌈(T/100)^(2/9)⌉ ≈ 1 (23).

The model was Yt=β_0_ + β_1_·time + β_2_·INT1 + β_3_·time_after_INT1 + β_4_·INT2 + β_5_·time_after_INT2 + εt (21). The parameters are interpreted as follows: β_0_ is the intercept, that is, the model-implied value of Yt at the origin of the time index; β_1_ is the pre-intervention baseline slope; β_2_ is the immediate level shift at the first intervention point, relative to the extrapolated baseline trend; β_3_ is the incremental slope change for the second segment, whose absolute slope is β_1_ + β_3_; β_4_ is the immediate level shift at the second intervention point, relative to the extrapolated segment-2 trend; and β_5_ is the incremental slope change for the third segment, whose absolute slope is β_1_ + β_3_ + β_5_. Accordingly, β_3_ and β_5_ should not be interpreted as the absolute slopes of segments 2 and 3. Because the SGK Statistical Yearbooks report counts on a calendar-year basis, the March 2020 COVID-19 breakpoint was operationalized as a calendar-year cutoff, so that both INT2 and time_after_INT2 equal 1 from t=2020 and the whole of 2020 is treated as a post-intervention year. Full ITS regression coefficients are reported in the supplementary material (https://www.sjweh.fi/article/4331) table S4. Analyses were performed in Python 3.12 (statsmodels 0.14, pandas 2.2); P-values <0.05 were considered statistically significant. We confined our interpretation to relationships directly observable in the yearbook data. Model goodness-of-fit was evaluated using the coefficient of determination (R^2^), which represents the proportion of variance in the occupational injury and recognized occupational disease rates explained by the segmented regression models. Reporting followed the Reporting of studies Conducted using Observational Routinely-collected health Data (RECORD) statement (25).

### Ethics

All data are publicly published aggregate counts with no identifiable individual information; no formal ethics review was required.

## Results

### Cohort overview and 18-year baseline transition

Between 2007 and 2024, the actively insured employee population doubled from approximately 8.5 million to 17.0 million (table 1). The proportion of women in the insured workforce rose from approximately 24% to 32%. Reported OI cases rose 9.1-fold from 80 602 to 733 646; recognized OD cases declined from 1208 to 888. The OI/OD ratio increased monotonically from 67 to 826 – a 12.3-fold widening of the gap between injury detection and disease recognition. The proportion of recognized OD occurring in women rose 28-fold from 0.9% to 25.5%, with the largest single-year increase between 2019 and 2020 (8.4% to 20.3%). However, this aggregate convergence conceals marked diagnostic asymmetry, detailed below: female visibility in OD recognition was achieved primarily through categories aligned with feminized service-sector and chemical-exposure work, while remaining negligible in pneumoconioses.

**Table 1:** Baseline characteristics of the Turkish insured employee surveillance cohort, 2007 versus 2024, with sex-stratified diagnostic transitions (Social Security Law 5510, Article 4-1/a coverage). [OD=occupational disease; ICD-10=International Classification of Diseases, 10^th^ revision; J62.8=silicosis sub-code; G62.2=polyneuropathy due to other toxic agents; OHS=occupational health and safety; NA=not applicable (category did not exist in the pre-2014 classification); pp=percentage points.] Sources: Social Security Institution Statistical Yearbooks 2007 and 2024 (20). Female workforce share estimated from Turkish Statistical Institute Labor Force Statistics (37).

Characteristic	2007	2024	18-year change
**Insured population**			
Total insured employees (N)	8 505 390	17 000 000	+99.9%
Women (% insured workforce)	~24	~32	+8 pp
**Surveillance outputs**			
Reported OI cases (N)	80 602	733 646	×9.1 fold
Recognized OD cases (N)	1208	888	−26.5%
OI deaths (N)	1043	1897	+81.9%
OD deaths (N)	1	3	—
**Surveillance rates**			
OI rate per 100 000 insured	948	4 316	×4.6 fold
Recognized OD rate per 100 000 insured	14.2	5.2	−63.4%
OI/OD ratio	67	826	×12.3 fold
OI case-fatality ratio per 1000 cases	12.9	2.6	−79.8%
**Sex distribution of cases**			
Women (% of OI)	5.1	24.1	+19.0 pp
Women (% of recognized OD)	0.9	25.5	+24.6 pp
Male-to-female ratio in OI	18.6	3.2	−83%
Male-to-female ratio in recognized OD	109	2.9	−97%
**Sex-stratified diagnostic ** **composition (2018 → 2024)**			
Women (% of respiratory OD)	1.2 (2018)	4.3	+3.1 pp
Women (% of musculoskeletal OD)	20.4 (2018)	34.0	+13.6 pp
Women (% of nervous-system OD)	24.2 (2018)	75.5	+51.3 pp
G62.2 toxic polyneuropathy among women (N)	0 (2018)	26	New cluster (2024)
**Diagnostic composition of ** **recognized OD**			
Silicosis (J62.8) (N)	354 (2008)	91	−74%
ICD-10 Z (unspecified, %)	NA	45	—
**Policy and surveillance context**			
OHS Law 6331 status	Not in force	In force since 12/2012	—
Active screening for high-risk cohorts	0	0	—
National OD reference hospitals (N)	3	3	—

### Eighteen-year trends and interrupted time series analysis

Three regime shifts are visible in the time series (figure 1A, 1B; supplementary tables S1 and S4). During 2007–2012, both rates declined: OI from 948 to 627 per 100 000 (APC −1.5%/year), OD from 14.2 to 3.3 (APC −20.0%/year). After the OHS Law (2013–2019), OI rose to 2 864 per 100 000 (APC +14.1%/year) while OD rebounded modestly to 7.4 (APC +20.7%/year, from a much lower base). In 2020–2024, OI climbed to 4 316 per 100 000 (APC +17.5%/year), but the OD rate decreased to 5.2 (APC −0.6%/year). The OI/OD ratio rose from 423 in 2020 to 826 in 2024.

Segmented regression with two intervention points showed striking asymmetry between the surveillance streams (figure 1C, 1D; supplementary table S4). Additionally, the derived absolute slopes for each segment, β_1_ for 2007–2012, β_1_ + β_3_ for 2013–2019, and β_1_ + β_3_ + β_5_ for 2020–2024, are reported alongside the raw regression coefficients in supplementary table S4. For OI rate, the 2012 OHS Law produced an immediate level change of +584 per 100 000 (95% CI 393–775; P<0.001, relative to the baseline trend) and a sustained slope change of +323/year (P<0.001; derived absolute slope for segment 2=+257.5/year). The COVID-19 disruption produced a level reduction of −551 per 100 000 (P<0.001; relative to the extrapolated pre-pandemic trend, defined as the segment-2 trend [β_1_ + β_3_] extrapolated to 2020) followed by a slope increase of +148/year (P=0.001; derived absolute slope for segment 3=+405.5/year), with rates returning to the pre-pandemic trend within two years (R^2^=0.992, adjusted R^2^=0.988). In contrast, the OD rate showed no significant level change at 2012 (β_2_=−1.11; 95% CI −3.31–1.10; P=0.324), although it experienced a significant slope change of +2.29/year (P<0.001; derived absolute slope for segment 2=+0.77/year). The 2020 intervention produced a non-significant level change (β_4_=+0.40; P=0.597; relative to the segment-2 trend [β_1_ + β_3_] extrapolated to 2020) followed by a significant negative slope change for OD (β_5_=−1.20/year; P<0.001; derived absolute slope for segment 3=−0.43/year; R^2^=0.654, adjusted R^2^=0.465).

**Figure 1:**
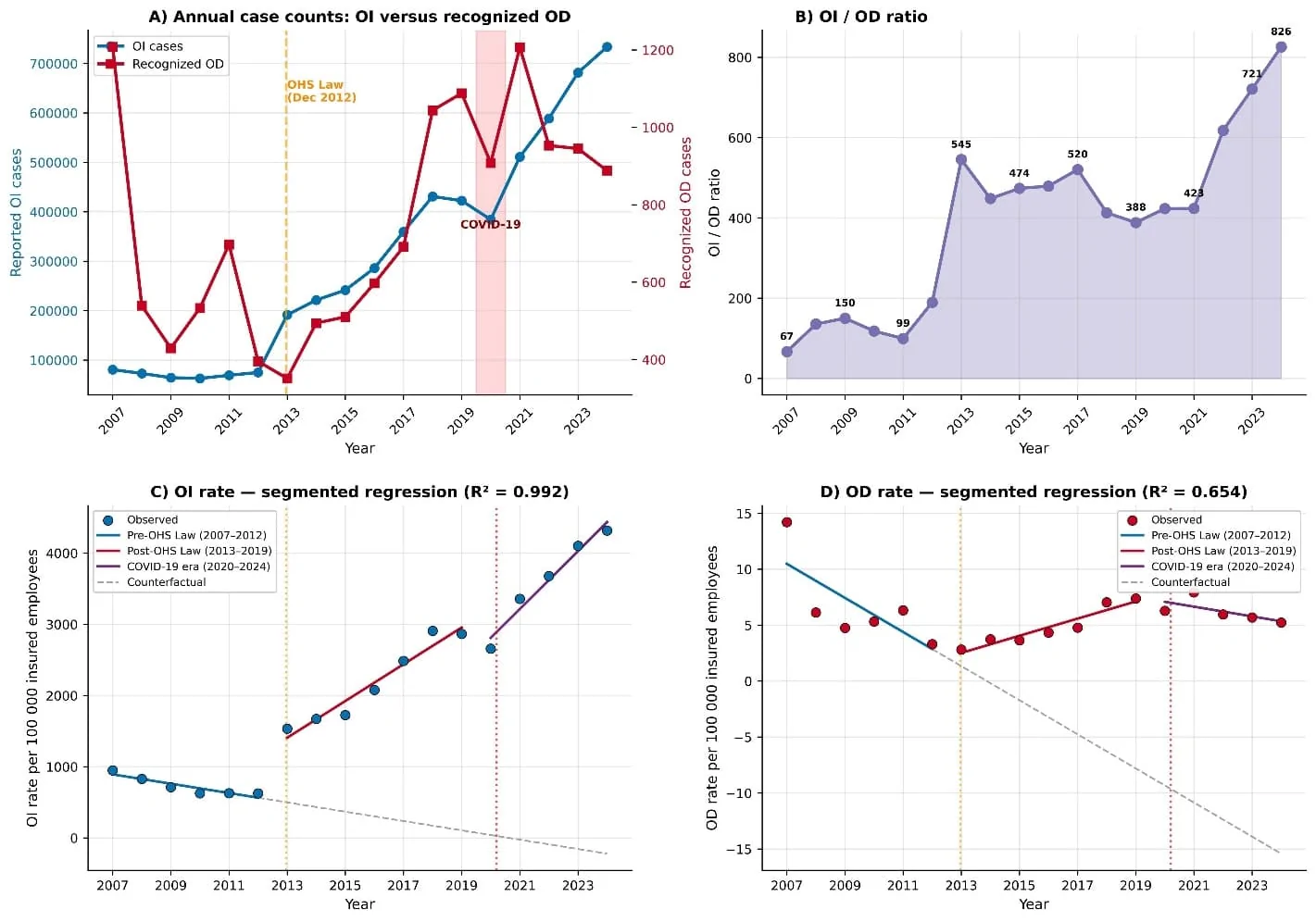
Surveillance trends and interrupted time series analysis, Türkiye 2007–2024. Panel A: annual case counts of OI (blue circles, left axis) versus recognized OD (red squares, right axis); the orange dashed line indicates the December 2012 OHS Law, and the red shaded band indicates the COVID-19 lockdown period. Panel B: OI/OD ratio from 2007 to 2024. Panel C: segmented regression of OI rate per 100 000 insured employees with two intervention points; the gray dashed line shows the counterfactual extrapolation of the pre-2012 trend. Panel D: segmented regression of OD rate. Vertical dotted lines mark intervention dates; specifically in panels C and D, the orange dotted line indicates the December 2012 OHS Law, and the red dotted line indicates the March 2020 COVID-19 onset. The red shaded band in Panel A highlights the acute lockdown and peak restriction phase (2020–2021), whereas Panels C and D illustrate the full post-pandemic analytical segment (2020–2024) as modeled in the interrupted time series analysis. In Panel D, the lower tail of the counterfactual projection extending below zero represents a mathematical model extrapolation rather than observed data, reflecting the theoretical continuation of the prior trend. [OI=occupational injury; OD=compensation-recognized occupational disease; OHS=occupational health and safety; R^2^=coefficient of determination.] The OD response shows no significant level change at 2012 (P=0.324). Source: Social Security Institution Statistical Yearbooks 2007–2024 (Social Security Law 5510, Article 4-1/a coverage).

### Sex disaggregation and mortality

Female representation in the OI stream rose from 5.1% (2007) to 24.1% (2024) (figure 2A); the male-to-female ratio dropped from 18.6 to 3.2. Women’s share of recognized OD rose from 0.9% to 25.5% (figure 2B), with the largest single-year increase between 2019 and 2020 (8.4% to 20.3%); the yearbook tables do not provide industry- or exposure-level attribution. Annual occupational injury deaths totaled approximately 25 700 across the 18-year period; OD-related deaths cumulatively totaled 84. The case-fatality ratio declined from 12.9 per mille (2007) to 2.6 (2024), peaking at 24.6 per mille in 2011 before the OHS Law (figure 2C).

**Figure 2:**
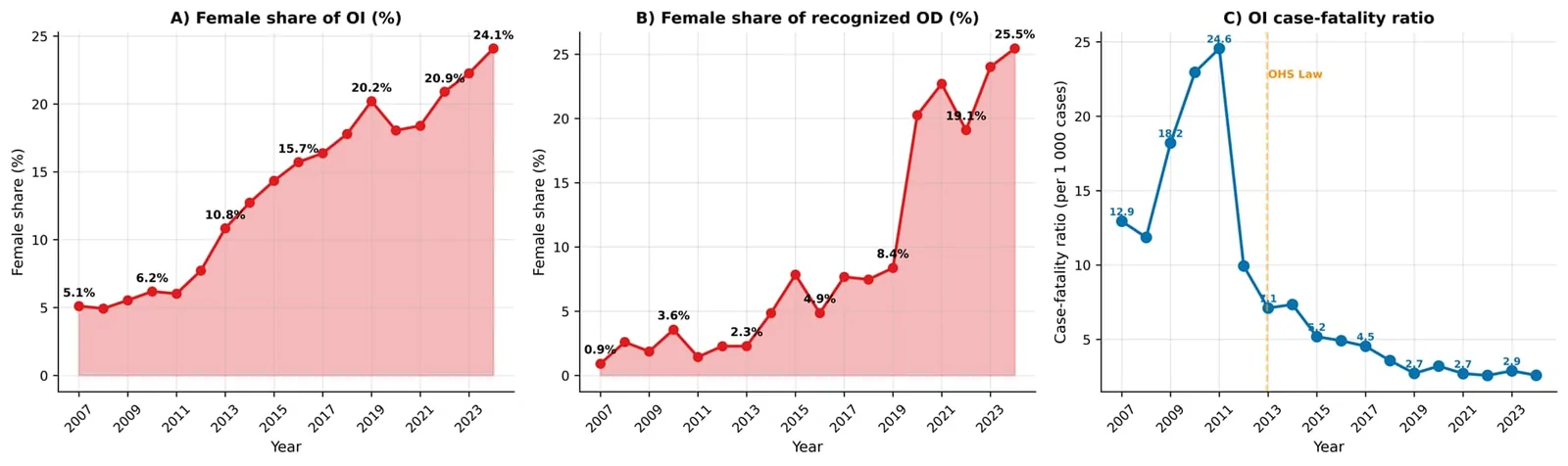
Sex-disaggregated trends and mortality, Türkiye 2007–2024. Panel A: proportion of occupational injuries occurring in women (%), rising from 5.1% in 2007 to 24.1% in 2024. Panel B: proportion of recognized occupational disease occurring in women (%), rising from 0.9% to 25.5%, with the largest single-year jump between 2019 and 2020 (8.4% to 20.3%). Panel C: occupational injury case-fatality ratio (deaths per 1 000 reported OI cases), declining from 12.9 per mille (2007) to 2.6 per mille (2024); the orange dashed line indicates the December 2012 OHS Law. Abbreviations: OI = occupational injury; OD = compensation-recognized occupational disease; OHS = Occupational Health and Safety. Source: Social Security Institution Statistical Yearbooks 2007–2024.

### Sex-stratified diagnostic-category trajectories

Disaggregation by sex within ICD-10 categories revealed marked heterogeneity (supplementary table S2). Respiratory OD – dominated by silicosis, asbestosis, and coal worker pneumoconiosis – remained overwhelmingly male: 96% of 2024 cases. Female respiratory OD cases (N=10 in 2024) comprised 9 occupational asthma (J45.0/J45.1/J45.9) and 1 asbestosis (J61); no female silicosis (J62.8) was recorded in 2018–2024.

Three diagnostic categories showed marked feminization (figure 3). Musculoskeletal disorders rose from 49 cases (20% female) in 2018 to 100 cases (34% female) in 2024 (figure 3A). Within the female increase, dominant ICD-10 sub-codes were M70.0 (chronic crepitant synovitis of hand and wrist; 13 of 34 female cases in 2024), M77.1 (lateral epicondylitis; 9), M77.0 (medial epicondylitis; 4), and M65.04 (tendon sheath infection; 5) – all upper-extremity repetitive-motion disorders. The most pronounced sex shift appeared in the nervous-system category (G chapter), from 33 cases (24% female) in 2018 to 53 cases (75% female) in 2024 (figure 3B). Skin diagnoses, smaller in absolute number, also showed rising female participation (figure 3C). Sectoral attribution cannot be established from these data alone.

**Figure 3:**
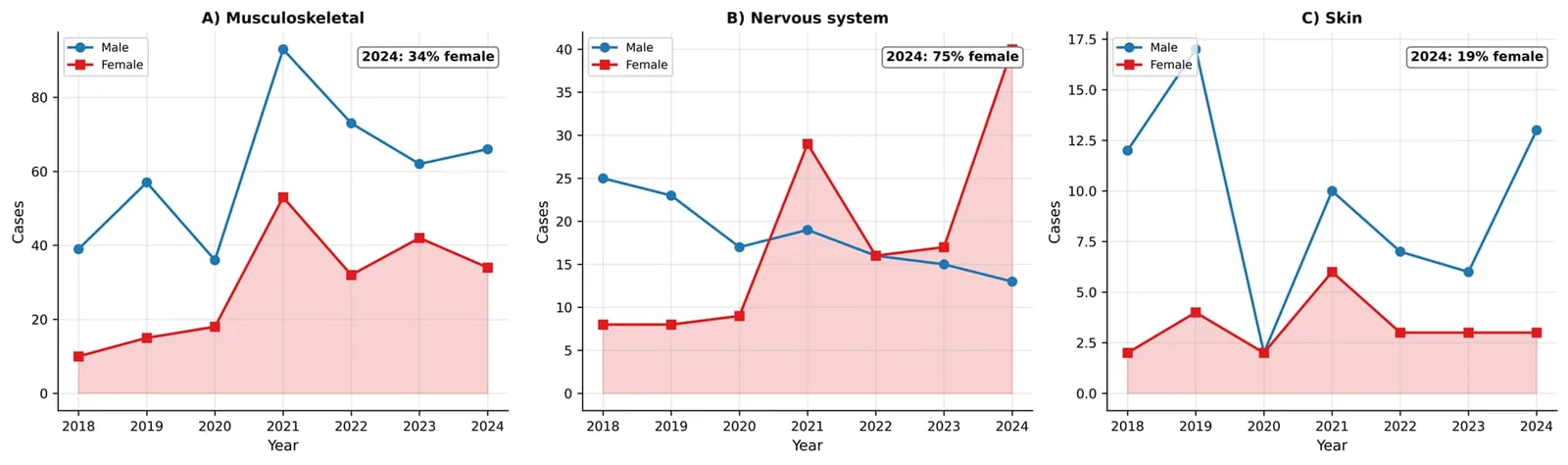
Sex-disaggregated trends in three ICD-10 diagnostic categories with marked female participation, Türkiye 2018–2024. Panel A: musculoskeletal disorders, rising from 20% female (2018) to 34% female (2024). Panel B: nervous-system disorders, with female share rising from 24% (2018) to 75% (2024). Panel C: skin disorders, with female share rising from 14% (2018) to 19% (2024). Blue circles indicate male cases; red squares indicate female cases. Abbreviations: ICD-10 = International Classification of Diseases, 10th revision; OD = occupational disease. Source: Social Security Institution Statistical Yearbooks 2017–2024 (Tablo 3.1.10, Social Security Law 5510 Article 4-1/a coverage).

### Sub-diagnoses driving the female nervous-system increase

Sub-code analysis of the G chapter revealed two distinct features (supplementary table S5). First, ICD-10 G62.2 polyneuropathy due to other toxic agents accounted for 26 of 40 female nervous-system cases in 2024 (65%). G62.2 was absent from female recognized OD throughout 2018–2022, appeared with 4 cases in 2023, and surged to 26 cases in 2024; corresponding male G62.2 counts remained low (0–4/year). The yearbook does not specify the industries or chemical agents involved; this concentration in a single sub-code is most consistent with one or more cluster recognitions of chemical-exposure neuropathy among female workers in 2023–2024. Second, a temporally distinct cluster was visible in 2021: G56.2 lesion of ulnar nerve rose from 2 female cases in 2020 to 22 in 2021, then declined to 1 in 2024. Female recognized OD across all years shows no recorded cases under ICD-10 chapter F (mental and behavioral disorders); burnout-related diagnoses do not appear in either sex. As an additional observation, no COVID-19 case (ICD-10 codes U07–U09) appears in SGK compensation-recognized infectious OD across 2020–2024; the recognized infectious sub-codes were limited to tuberculosis, brucellosis, Q fever, and hemorrhagic fever (annual counts 1–8).

### Sectoral distribution

In 2024, the highest absolute OI counts occurred in building construction, food and beverage services, food manufacturing, and fabricated metals (supplementary table S3). The highest OD/OI ratios occurred in manufacture of other non-metallic mineral products (NACE 23: glass, ceramics, cement; 3.1 per mille) and basic metals (NACE 24; 2.2 per mille). Building construction recorded only 27 OD cases against 50 429 injuries (0.5 per mille).

## Discussion

Eighteen years of national surveillance data demonstrate that Türkiye’s 2012 OHS Law had a robust, sustained impact on occupational injury reporting – an immediate level shift of +584 cases per 100 000 insured and a sustained slope increase of +323/year – but no statistically significant immediate change in compensation-recognized OD. The OI/OD ratio rose from 67 to 826, a 12-fold widening of the gap between injury detection and disease recognition that is unlikely to be explained by changes in workplace exposure alone. Within this gap, our central finding is the differentiated pattern of female occupational disease recognition that emerged over the period.

The aggregate convergence of women’s share of cases toward 25% in both streams – from 5% (OI) and 1% (OD) – reflects real growth of female participation in formal employment, expanded coverage of female-dominated sectors, and improved registration practices. However, it masks marked diagnostic asymmetries. Women became visible in compensation-recognized OD primarily through musculoskeletal disorders driven by upper-extremity repetitive-motion sub-codes and through chemical-exposure neuropathy (G62.2), but remained scarcely represented in pneumoconioses, accounting for fewer than 5% of cases despite documented mixed-exposure pathways (6). Systematic gender bias in occupational disease recognition has been documented internationally (7–9). Camp et al (10) reviewed how female occupational lung disease is underdetected because exposure assessments and clinical suspicion are calibrated for male-dominated occupations. Messing and colleagues (7, 11) have shown across jurisdictions that workers’ compensation systems systematically reject a higher proportion of female applications and that women face longer diagnostic delays than men with comparable exposures. Criado Perez (12) has framed this as a structural problem of male-calibrated exposure thresholds and protective-equipment standards.

Several contextual explanations must therefore be considered alongside possible diagnostic bias. The rise in women’s formal labor-force participation, expansion of female-dominated service and manufacturing work, and improved registration can increase women’s share of recognized cases even if recognition probabilities remain unequal. Abrupt code-specific increases, such as G56.2 in 2021 and G62.2 in 2023–2024, may also reflect workplace clusters or administrative batch completion rather than population-wide incidence. Moreover, foreign workers with valid permits are generally insured and included when registered, whereas workers in irregular or otherwise unregistered employment are absent. Because SGK does not publish nationality-by-sex tables, application and rejection denominators, or case-level exposure and occupation linkages, the contribution of migrant-workforce composition and sex-specific acceptance cannot be quantified. The observed asymmetries are therefore compatible with barriers documented in other compensation systems but do not, by themselves, establish diagnostic bias.

A particularly instructive Turkish example is Kerget et al (6): a female worker employed as a controller in a denim sandblasting facility developed silicosis through bystander exposure but was not diagnosed for ten years. Her clinicians repeatedly failed to elicit an occupational history because sandblasting was framed as exclusively male work, and she underwent multiple invasive procedures under alternative diagnoses – most prominently sarcoidosis – before silicosis was recognized. This case illustrates the diagnostic under-recognition that female workers in mixed-exposure environments encounter: even when present in dusty workplaces in non-primary roles (controller, cleaner, packaging assistant), occupational history-taking is shaped by the gendered framing of who counts as exposed. The 98.9% male share among Australian engineered-stone silicosis claimants (19) and the absence of female silicosis cases in our 2018–2024 SGK dataset are most parsimoniously interpreted as a combination of real differential exposure and systematic under-recognition of female cases by diagnostic and compensation pathways.

The patterns where women did become visible are consistent with this framework. Female workers are disproportionately concentrated in occupations involving cleaning chemical exposure (26), repetitive upper-extremity work in healthcare and manufacturing (27), and chemical solvent exposure in electronics and small-component assembly (28). Our finding of upper-extremity repetitive-motion disorders driving the female musculoskeletal increase, and the G62.2 toxic polyneuropathy cluster in 2023–2024, is biologically consistent with these gendered exposure profiles. The G62.2 cluster is particularly notable: absent throughout 2018–2022, 4 cases in 2023, then 26 in 2024 (65% of all female nervous-system cases that year). The annual concentration in a single sub-code is most consistent with one or more cluster recognitions of chemical-exposure neuropathy among female workers; sectoral attribution requires linkage to non-public administrative datasets. Recognition of such clusters depends on physician suspicion and Health Committee acceptance, both subject to documented gender bias (7, 9). Strengthening Turkish surveillance therefore requires not only structural reform of the compensation pathway but also gender-sensitive training of occupational physicians and revision of the diagnostic code stock to better capture the morbidity patterns observed in female workers.

Three observations about the trajectory of the two surveillance streams deserve emphasis. First, the divergence after 2020: recognized OD fell from 1207 cases (7.9 per 100 000) in 2021 to 888 cases (5.2) in 2024 – a decline of approximately 34%, corresponding to a significant negative slope change (β_5_=−1.20/year; P<0.001). Rather than a singular deterioration in case recognition, this decline may reflect several concurrent mechanisms: reduced throughput in authorized Health Committees due to healthcare system strain, altered application behaviors as workers avoided hospitals, and genuine reductions in hazardous workplace exposures resulting from widespread remote-work transitions. Given that workplace exposure to silicogenic dusts, ergonomic load, and chemical agents did not contract over this period, the most parsimonious interpretations are that fewer suspected diseases are being notified, or that notified cases may be less likely to complete the SGK recognition process (typically expressed as a meslek hastalığı kazanılmış güç kaybı oranı below the 10% compensation threshold). A true decline in clinical incidence is, in our view, the least likely of these alternatives, given the unchanged exposure structure. Second, the post-2013 OI slope (+323/year) substantially exceeds the post-2013 OD slope (+2.29/year); the 2020 OI dip recovered within two years to surpass pre-pandemic levels, with the post-pandemic slope even steeper. This rebound contrasts sharply with OD and is consistent with OI reporting reflecting an employer-driven, supply-side process that responds rapidly to resumed economic activity, whereas OD recognition reflects worker-initiated, multi-stage processes that remain dampened. Third, the decline in OI case-fatality ratio from 12.9 to 2.6 per mille (approximately 80% reduction) cannot be attributed to workplace safety alone: the denominator expanded ninefold while deaths grew less than twofold. Three contributing mechanisms are plausible: a rise in the reporting of minor injuries that would not have entered surveillance before 2013; modest improvements in workplace safety; and improvements in pre-hospital trauma care and emergency response that have raised survival following severe injuries (29).

Our analysis must be interpreted in light of Türkiye’s compensation-based surveillance design, which contrasts sharply with diagnosis-based systems such as the Finnish register of occupational diseases (FROD) and Germany’s statutory accident insurance system BGB) (4, 30). The gap between clinically diagnosed and compensation-recognized OD has been documented internationally as ranging from 2- to 10-fold (24). In Türkiye, the gap appears larger: applying European clinical incidence (approximately 120 OD per 100 000 insured (32)) to Türkiye’s 2024 insured population (approximately 17 million) implies roughly 20 400 expected clinical OD cases, against an observed compensation-recognized count of 888. This is a benchmark of the compensation-recognition filter, not a prevalence estimate. The OHS Law expanded employer-side reporting obligations and modernized digital infrastructure, which act primarily on injury report supply. OD recognition, by contrast, depends on demand-side capacity that the law did not address: physicians who recognize disease–exposure causality, coordinated medical surveillance for latent disease, accessible application pathways, and Health Committee evaluation infrastructure for latent conditions.

The decline in silicosis recognitions – 354 in 2008 to 91 in 2024 – deserves particular attention. The 2008–2012 decline is biologically plausible following the 2009 ban on denim sandblasting (16, 17). However, the post-2012 continued decline is incongruent with the global context: since 2018, large case clusters of accelerated silicosis associated with engineered-stone work have been described in Australia, the United States, Spain, and Israel (18, 19, 32). In Victoria, Australia, 32-year compensation data showed 536 silicosis claims with a male share of 98.9% (19). Türkiye is among the largest engineered-stone producers and consumers in the region; severe accelerated silicosis progresses to disability and death within 5–10 years and is unlikely to present subtly. If such cases exist in Türkiye, they are not entering the compensation-recognition pathway. The complete absence of female silicosis cases over the 2018–2024 study period, against Kerget et al’s (6) demonstration that female bystander exposure does produce silicosis in Turkish workplaces, further reinforces the inference that mixed-exposure female cases are systematically failing to reach recognition.

Notably, no SARS-CoV-2 case (ICD-10 U07–U09) appears in SGK-recognized infectious OD during 2020–2024; Italian data have shown that recognition of occupational COVID-19 requires substantial protocol adaptation and lead times in compensation systems (33).

Türkiye’s compensation-recognized OD rate in 2024 (5.2 per 100 000 insured) is approximately one twenty-fourth of the European Union average for clinical OD incidence (30). The South Korean experience offers an instructive contrast: statutory special health examinations for dust-exposed workers, recently supplemented by hospital-based sentinel surveillance, sustained recognition of 1679 pneumoconiosis cases in 2022 alone (34).

Strengths of this study include the longest continuous time series of Turkish OI and recognized OD published to date, covering three policy regimes; the framing of Turkish OD surveillance as a compensation-based filter within the international workers’ compensation literature (31, 35); and sub-code-resolution sex-stratified analysis identifying specific signal sources within aggregate trends. Limitations include the following. First, the 2014 change in diagnostic classification constrains comparison of pre- and post-2014 categorical distributions. However, this grouping change occurred one calendar year after the 2013 ITS breakpoint; because the ITS outcome is total OD count rather than category-specific count, and because sub-code reporting was operative throughout 2007–2024, the 2013 OD level coefficient (β_2_=−1.11; 95% CI −3.31–1.10; P=0.324) is unlikely to be driven by the coding transition. This is corroborated by visual inspection of figure 1D showing no incremental step at 2014. Second, the 2007 OD count (1 208) is anomalously high and the 2012 mortality count (744) is anomalously low; we did not adjust for either. The 2007 peak could plausibly result from administrative backlog clearance following the institutional merger into SGK, or other reporting-regime discontinuities. Similarly, the 2012 mortality trough might represent an administrative reporting artifact – potentially caused by data-visibility gaps during the transition to the İSG-KATİP digital platform under the new OHS Law – rather than a genuine short-term safety improvement. Third, our analysis covers only insured workers; approximately 30% of Türkiye’s workforce is informally employed (36) and is not captured by this surveillance system – a population disproportionately female and concentrated in agriculture, domestic service, and small-scale manufacturing (37), which compounds the under-recognition of female disease. Furthermore, diagnostic detail tables count distinct codes per case, which may exceed summary-table totals. Moreover, the SGK published data do not link cases to specific industries, exposures, or locations at the case level; sectoral attribution of diagnostic clusters requires linkage to non-public administrative datasets. Finally, the annual aggregation of the SGK data inherently masks within-year dynamics: the March 2020 COVID-19 intervention was operationalized at the calendar-year level, treating 2020 entirely as a post-intervention year and thereby folding the first two pre-pandemic months into the annual aggregate.

The yearbooks do not report the total number of occupational-disease applications, rejected cases, reasons for rejection, processing times, or nationality-by-sex distributions. Consequently, the available data do not permit calculation of an acceptance rate or assessment of whether rejection patterns and the availability and completeness of supporting medical and workplace-exposure documentation differ by sex, occupation, or diagnosis.

Six policy directions follow from our findings, with explicit attention to female occupational disease visibility. First, the compensation-based design should be supplemented by a parallel diagnosis-based notification stream requiring notification at the point of clinical diagnosis, independent of compensation. Second, active medical surveillance should be implemented for high-silica-exposure occupations including engineered-stone workers, with mandatory annual spirometry and high-resolution computed tomography for symptomatic workers, and inclusion criteria explicitly extended to women in mixed-exposure roles such as controllers, cleaners, and packaging assistants (6, 19). Third, a chemical-exposure registry should be established to permit tracing of cluster recognitions – such as the 2024 G62.2 surge – back to source workplaces, with particular attention to female-dominated sectors. Fourth, regional occupational disease hospital capacity should be expanded beyond the three existing centers. Fifth, the notification obligation should be extended to neurologists, dermatologists, otolaryngologists, and physical medicine specialists, and gender-sensitive training in occupational history-taking should be incorporated into postgraduate medical education (7, 10). Sixth, the ICD-10 Z category, which absorbed 45% of 2024 OD recognitions, should be subjected to detailed file-level audit with sex-disaggregated reporting, and Health Committee criteria should be reviewed periodically to accommodate emergent occupational exposures.

In summary, Türkiye’s 2012 OHS Law produced a quantifiable, sustained increase in workplace injury reporting but had no detectable effect on compensation-recognized OD. The divergence between sustained OI growth and OD decline since 2020 indicates that the surveillance gap is actively widening, with the most plausible explanation residing in the notification and compensation-recognition pathway rather than in changes in underlying exposure. Within this gap, the 28-fold rise in women’s share of OD represents a meaningful advance but conceals an underlying asymmetry: women became visible primarily in feminized service-sector and chemical-exposure diagnostic categories – musculoskeletal repetitive-motion disorders, toxic polyneuropathy, occupational asthma – while remaining scarcely represented in pneumoconioses despite established mixed-exposure pathways. The persistence of 96% male representation in respiratory OD indicates that the aggregate convergence of women’s share toward 25% reflects sectoral expansion rather than resolution of gender bias. Closing this surveillance gap will require diagnosis-stage notification, active surveillance programs with explicit gender-sensitive inclusion criteria, expanded notifier mandates, a chemical-exposure registry, and updated diagnostic categories that capture emerging occupational hazards in feminized workforces.

## Supplementary material

Supplementary material 1 Supplementary material

## Data Availability

Raw data are publicly available at https://www.sgk.gov.tr/Istatistik/Yillik/. Processed analytical datasets and analysis code are available from the corresponding author on request.
