# Supplementary material for: Asymmetric impact of the 2012 Occupational Health and Safety Law on occupational injury and disease reporting in Türkiye: An interrupted time series analysis with emphasis on emerging female occupational disease patterns
Source: Scand J Work Environ Health. 2026 Oct 26;52(6):744–55. doi: 10.5271/sjweh.4331 (PMC13738665; doi:10.5271/sjweh.4331)
Supplement: Supplementary file 1 [file SJWEH-52-6-744-s001.pdf]

Asymmetric impact of the 2012 Occupational Health and Safety Law on occupational injury and disease reporting in Türkiye: An interrupted time series analysis with emphasis on emerging female occupational disease patterns[1]

by Funda Ekimci Deniz, MD, PhD,[2] Zehra Nur Töreyin, MD, PhD, Metin Akgün, MD, PhD

1. Supplemental material
2. Correspondence to: Funda Ekimci Deniz, Department of Family Medicine, Faculty of Medicine, Aydın Adnan Menderes University, Aydın Adnan Menderes Üniversitesi, Tıp Fakültesi Dekanlığı, Merkez Kampüs, 09100 Efeler – Aydın, Türkiye. [E-mail: fedeniz@adu.edu.tr] ORCID: 0000-0002-8308-5024

Contents

Supplementary Table S1. Annual occupational injury and recognized occupational disease data, 2007–2024.

Supplementary Table S2. Sex-stratified occupational disease in major diagnostic categories, 2018 vs 2024.

Supplementary Table S3. Top 15 economic sectors by occupational injury count, 2024.

Supplementary Table S4. Interrupted time series regression results (2012 OHS Law and 2020 COVID-19 intervention points).

Supplementary Table S5. ICD-10 G-chapter sub-code breakdown of nervous-system occupational disease by sex and year, 2018–2024.

Supplementary Table S1. Annual occupational injury and recognized occupational disease cases, deaths, and incidence rates per 100 000 insured employees, Türkiye 2007–2024.

Year	OI cases	OD cases	OI deaths	OD deaths	OI rate[1]	OD rate[1]	OI/OD ratio	CFR[2]
2007	80 602	1 208	1 043	1	948	14.2	67	12.9
2008	72 963	539	865	1	829	6.1	135	11.9
2009	64 316	429	1 171	0	712	4.8	150	18.2
2010	62 903	533	1 444	10	627	5.3	118	23
2011	69 227	697	1 700	10	628	6.3	99	24.6
2012	74 871	395	744	1	627	3.3	190	9.9
2013	191 389	351	1 360	0	1533	2.8	545	7.1
2014	221 366	494	1 626	0	1672	3.7	448	7.3
2015	241 547	510	1 252	0	1725	3.6	474	5.2
2016	286 068	597	1 405	0	2077	4.3	479	4.9
2017	359 653	691	1 633	0	2484	4.8	520	4.5
2018	430 985	1 044	1 541	0	2907	7	413	3.6
2019	422 463	1 088	1 147	0	2864	7.4	388	2.7
2020	384 262	908	1 231	5	2657	6.3	423	3.2
2021	511 084	1 207	1 382	35	3357	7.9	423	2.7
2022	588 823	953	1 517	8	3677	6	618	2.6
2023	681 401	945	1 966	0	4101	5.7	721	2.9
2024	733 646	888	1 897	3	4316	5.2	826	2.6

Abbreviations: OI = occupational injury; OD = compensation-recognized occupational disease; CFR = case-fatality ratio. [1]Rate per 100 000 insured employees per year. [2]Deaths per 1 000 reported OI cases (per mille). Source: Social Security Institution Statistical Yearbooks 2007–2024 (reference 20 in main manuscript).

Supplementary Table S2. Sex-stratified counts of recognized occupational disease in major ICD-10 diagnostic categories, Türkiye 2018 versus 2024.

Diagnostic category	M 2018	F 2018	T 2018	Women, % 2018	M 2024	F 2024	Women, % 2024
**Respiratory (incl. pneumoconioses)**	735	9	744	1.2%	246	11	4.3%
**Z – Unspecified**	169	40	209	19.1%	288	115	28.5%
**Eye/Ear (incl. NIHL)**	87	8	95	8.4%	88	11	11.1%
**Musculoskeletal**	39	10	49	20.4%	66	34	34.0%
**Nervous system**	25	8	33	24.2%	13	40	75.5%
**Skin**	12	2	14	14.3%	13	3	18.8%
**Infectious**	3	4	7	57.1%	2	0	0.0%
Total (selected categories)	***1 070***	***81***	***1 151***	***7.0%***	***716***	***214***	***23.0%***

Abbreviations: M = male; F = female; T = total; ICD-10 = International Classification of Diseases, 10th revision; NIHL = noise-induced hearing loss. Bottom row in bold italic denotes column totals across the selected diagnostic categories. Source: SGK Statistical Yearbooks, Tablo 3.1.10. Diagnostic-detail tables count distinct diagnostic codes per case; column totals may exceed summary-table OD totals.

Supplementary Table S3. Top 15 economic sectors by occupational injury count, with corresponding recognized occupational disease count and OD/OI ratio, Türkiye 2024.

Rank	NACE Rev. 2.1 sector	OI 2024	OD 2024	OD/OI ratio (per mille)
1	Construction of buildings	50 429	27	0.5
2	Food and beverage service activities	41 393	20	0.5
3	Manufacture of food products	39 604	26	0.7
4	Manufacture of fabricated metal products	37 510	46	1.2
5	Accommodation	36 762	1	0.0
6	Retail trade	34 398	60	1.7
7	Manufacture of textiles	33 721	19	0.6
8	Human health activities	32 064	9	0.3
9	Warehousing and transport	28 798	20	0.7
10	Manufacture of basic metals	26 711	58	2.2
11	Civil engineering	26 106	6	0.2
12	Buildings services / cleaning	26 206	13	0.5
13	Manufacture of motor vehicles	24 082	61	2.5
14	Manufacture of other non-metallic minerals	21 695	67	3.1
15	Manufacture of rubber/plastics	20 430	31	1.5

Abbreviations: NACE = Statistical Classification of Economic Activities in the European Community; OI = occupational injury; OD = occupational disease. Manufacture of other non-metallic minerals (NACE 23: glass, ceramics, cement) shows the highest OD/OI ratio (3.1 per mille). Construction of buildings (Rank 1 in injury count) shows the lowest ratio (0.5 per mille) despite established silicosis and asbestosis risk. Source: Social Security Institution Statistical Yearbook 2024 (reference 20 in main manuscript).

Supplementary Table S4. Interrupted time series analysis of occupational injury and recognized occupational disease rates with two intervention points (December 2012 OHS Law; March 2020 COVID-19), Newey–West HAC robust standard errors, lag truncation = 1.

Parameter	OI rate (95% CI)	OD rate (95% CI)	OI/OD ratio (95% CI)	P (OI / OD / ratio)
**Intercept (β_0)**	957.6 (868–1047)	12.0 (7.1–16.9)	79.1 (36.0–122.1)	<0.001 / <0.001 / <0.001
**Pre-trend (β_1) per year**	−65.5 (−89 to −42)	−1.52 (−2.55 to −0.49)	+13.5 (+2.3 to +24.8)	<0.001 / 0.004 / 0.018
**2012 level change (β_2)**	+584 (393–775)	−1.11 (−3.31 to 1.10)	+377 (324–430)	<0.001 / 0.324 / <0.001
**2012 slope change (β_3)**	+323 (271–375)	+2.29 (1.24 to 3.34)	−31.2 (−48.0 to −14.4)	<0.001 / <0.001 / <0.001
**2020 level change (β_4)**	−551 (−790 to −312)	+0.40 (−1.09 to 1.90)	−143 (−229 to −56)	<0.001 / 0.597 / 0.001
**2020 slope change (β_5)**	+148 (63 to 233)	−1.20 (−1.51 to −0.89)	+128 (112 to 144)	0.001 / <0.001 / <0.001
**Absolute slope, segment 1 (2007–2012), = β_1**	−65.5 (−89 to −42)	−1.52 (−2.55 to −0.49)	+13.5 (+2.3 to +24.8)	<0.001 / 0.004 / 0.018
**Absolute slope, segment 2 (2013–2019), = $\beta_1 + \beta_3$**	+257.5 (+182 to +333)	+0.77 (−1.31 to +2.85)	−17.7 (−45.7 to +10.4)	<0.001 / 0.305 / 0.086
**Absolute slope, segment 3 (2020–2024), = $\beta_1 + \beta_3 + \beta_5$**	+405.5 (+245 to +566)	−0.43 (−2.82 to +1.96)	+110.3 (+66.3 to +154.4)	<0.001 / 0.575 / <0.001
Model R^2	***0.992***	***0.654***	***0.973***	—
Adjusted R^2	***0.988***	***0.465***	***0.958***	**---**

Abbreviations: OI = occupational injury; OD = recognized occupational disease; OHS = Occupational Health and Safety; HAC = heteroscedasticity- and autocorrelation-consistent; CI = confidence interval. All rates per 100 000 insured employees. β_2 tests immediate level change at 2013; β_3 tests sustained slope change after the 2012 OHS Law. β_4 and β_5 are analogous for the March 2020 COVID-19 disruption. Bottom row (in bold italic) summarizes model fit (R^2 and adjusted R^2).

Supplementa^{r}y Table S5. ICD-10 G-chapter sub-code breakdown of nervous-system occupational disease by sex and year, Türkiye 2018–2024 (number of recognized cases).

ICD-10 sub-code	Sex	2018	2019	2020	2021	2022	2023	2024
**G56.0 Carpal tunnel syndrome**	F	5	6	7	0	14	8	6
**G56.0 Carpal tunnel syndrome**	M	14	17	11	0	10	8	6
**G56.2 Lesion of ulnar nerve**	F	0	2	1	22	2	2	1
**G56.2 Lesion of ulnar nerve**	M	7	4	3	9	2	2	0
**G56.3 Lesion of radial nerve**	F	0	0	0	1	0	0	0
**G56.8 Other upper-limb mononeuropathies**	F	1	0	0	3	0	2	4
**G62.2 Polyneuropathy due to other toxic agents**	**F**	**0**	**0**	**0**	**0**	**0**	**4**	**26**
**G62.2 Polyneuropathy due to other toxic agents**	**M**	**0**	**0**	**0**	**3**	**0**	**0**	**4**
**G62.8 Polyneuropathy, other specified**	F	0	0	1	1	0	1	2
G chapter — total female	***F***	***8***	***8***	***9***	***29***	***16***	***17***	***40***
G chapter — total male	***M***	***25***	***23***	***17***	***19***	***16***	***15***	***13***
Women, % of nervous-system OD	—	***24%***	***26%***	***35%***	***60%***	***50%***	***53%***	***75%***

Abbreviations: F = female; M = male; ICD-10 = International Classification of Diseases, 10th revision; OD = occupational disease. Bold rows for G62.2 toxic polyneuropathy denote the dominant single-code driver of the 2024 female nervous-system increase; the bottom three rows (bold italic) show G-chapter totals and the proportion of cases occurring in women. Two clusters are visible: G56.2 lesion of ulnar nerve rose to 22 female cases in 2021 and returned to baseline; G62.2 polyneuropathy due to other toxic agents was absent throughout 2018–2022, surfaced with 4 cases in 2023, and surged to 26 cases in 2024 (65% of all 2024 female nervous-system cases). Source: SGK Statistical Yearbooks, Tablo 3.1.10.
